# Avelumab first‐line maintenance in advanced urothelial carcinoma: Complete screening for prognostic and predictive factors using machine learning in the JAVELIN Bladder 100 phase 3 trial

**DOI:** 10.1002/cam4.7411

**Published:** 2024-06-24

**Authors:** Juliane Manitz, Aslihan Gerhold‐Ay, Pascal Kieslich, Parantu Shah, Thomas Mrowiec, Karin Tyroller

**Affiliations:** ^1^ EMD Serono Billerica Massachusetts USA; ^2^ The healthcare business of Merck KGaA Darmstadt Germany

**Keywords:** biomarkers, clinical cancer research, clinical trials, prognostic factor, urothelial

## Abstract

**Background:**

Avelumab first‐line (1 L) maintenance is a standard of care for advanced urothelial carcinoma (aUC) based on the JAVELIN Bladder 100 phase 3 trial, which showed that avelumab 1 L maintenance + best supportive care (BSC) significantly prolonged overall survival (OS) and progression‐free survival (PFS) vs BSC alone in patients who were progression free after receiving 1 L platinum‐containing chemotherapy. Here, we comprehensively screened JAVELIN Bladder 100 trial datasets to identify prognostic factors that define subpopulations of patients with longer or shorter OS irrespective of treatment, and predictive factors that select patients who could obtain a greater OS benefit from avelumab 1 L maintenance treatment.

**Methods:**

We performed machine learning analyses to screen a large set of baseline covariates, including patient demographics, disease characteristics, laboratory values, molecular biomarkers, and patient‐reported outcomes. Covariates were identified from previously reported analyses and established prognostic and predictive markers. Variables selected from random survival forest models were processed further in univariate Cox models with treatment interaction and visually inspected using correlation analysis and Kaplan–Meier curves. Results were summarized in a multivariable Cox model.

**Results:**

Prognostic baseline covariates associated with OS included in the final model were assignment to avelumab 1 L maintenance treatment, Eastern Cooperative Oncology Group performance status, site of metastasis, sum of longest target lesion diameters, levels of C‐reactive protein and alkaline phosphatase in blood, lymphocyte proportion in intratumoral stroma, tumor mutational burden, and tumor CD8+ T‐cell infiltration. Potential predictive factors included site of metastasis, tumor mutation burden, and tumor CD8+ T‐cell infiltration. An analysis in patients with PD‐L1+ tumors had similar findings to those in the overall population.

**Conclusions:**

Machine learning analyses of data from the JAVELIN Bladder 100 trial identified potential prognostic and predictive factors for avelumab 1 L maintenance treatment in patients with aUC, which warrant further evaluation in other clinical datasets.

## 1 INTRODUCTION

Urothelial cancer (UC) accounts for approximately 90% of all bladder cancers, and patients with unresectable locally advanced or metastatic UC (collectively termed advanced UC [aUC]) have a poor prognosis.^1^, ^2^, ^3^ Although ≈70% to 80% of patients with aUC have an objective response or stable disease with first‐line (1 L) platinum‐based chemotherapy, treatment responses are generally not durable and progression‐free survival (PFS) is short.^4^, ^5^, ^6^

In the phase 3 JAVELIN Bladder 100 trial (NCT02603432), avelumab maintenance + best supportive care (BSC) significantly prolonged overall survival (OS) and PFS versus BSC alone in patients with aUC without progression following 1 L platinum‐based chemotherapy.^7^, ^8^ After ≥2 years of follow‐up for OS in all patients, median OS measured from randomization (at start of maintenance, i.e., after chemotherapy) was 23.8 vs. 15.0 months, respectively (hazard ratio [HR], 0.76 [95% CI, 0.63–0.91]; two‐sided *p* = 0.004); median PFS was 5.5 vs. 2.1 months, respectively (HR, 0.54 [95% CI, 0.46–0.64]; two‐sided *p* < 0.0001).^8^ Analyses of patient‐reported outcomes showed similar findings between treatment arms, indicating that avelumab 1 L maintenance + BSC did not have a detrimental impact on quality of life compared with BSC alone.^9^ Following the results from the JAVELIN Bladder 100 trial, avelumab 1 L maintenance treatment was established as a standard of care for patients with aUC.^10^, ^11^, ^12^, ^13^

Although immune checkpoint inhibitors (ICIs) are an established part of the treatment landscape for aUC and various other cancers, only a subset of patients obtain durable responses to ICIs. Thus, the analysis of factors that can predict OS and identify which patients derive the greatest benefit from treatment has the potential to support clinical decision‐making. In patients with aUC receiving 1 L platinum‐based chemotherapy, previously validated risk factors predicting shorter OS include reduced Karnofsky performance status and the presence of visceral metastases (termed Bajorin risk factors).^14^ In analyses from the JAVELIN Bladder 100 trial, OS was longer in the programmed death ligand 1 (PD‐L1)–positive population than in the overall population in both treatment arms, suggesting that PD‐L1 status may be a prognostic factor for OS in a maintenance population.^7^ Additionally, subgroups with numerically shorter median OS in both treatment arms included those defined by Eastern Cooperative Oncology Group performance status (ECOG PS) and visceral metastases (including liver or lung metastases).^15^ However, in a comprehensive subgroup analysis, OS and PFS differences between arms in clinically relevant subgroups were generally consistent with results for the overall population, and no significant treatment‐by‐subgroup interaction (at the 0.05 level) was observed for any subgroup.^15^ Exploratory biomarker analyses from the JAVELIN Bladder 100 trial found that in the avelumab + BSC arm, OS benefit was positively associated with PD‐L1 expression by tumor cells, tumor mutational burden (TMB), CD8+ T cells, APOBEC mutation signatures, expression of genes underlying innate and adaptive immune activity, and the number of alleles encoding high‐affinity variants of activating Fc gamma receptors, highlighting that complex biologic pathways underlie treatment outcomes.^16^

A previous study aimed to identify potential biomarkers of treatment response using publicly available data from IMvigor210, a single‐arm phase 2 trial of atezolizumab (another anti–PD‐L1 immune checkpoint inhibitor) administered as 1 L monotherapy in cisplatin‐ineligible patients with aUC.^17^, ^18^ Among the variables examined, neoantigen burden and TMB had the greatest power for predicting treatment responses. A model combining neoantigen burden, TMB, ECOG PS, and gene expression signatures showed slightly increased predictive power. However, the authors concluded that combining the biomarkers did not improve response prediction significantly and that further analyses integrating independent biomarkers based on biological mechanisms are required.^18^

Comprehensive analyses integrating patient and disease characteristics in addition to molecular and tumor microenvironment biomarkers have the potential to generate hypotheses relevant for clinical decision‐making, meriting further evaluation. Machine learning is an emerging collection of advanced statistical methods that can be used to identify predictive and prognostic factors within clinical and biomarker data.^19^, ^20^, ^21^, ^22^ In this study, we performed machine learning using available datasets from the JAVELIN Bladder 100 trial.^7^ The objective was to identify patient, disease, or other characteristics that predict OS, which could help identify subpopulations of patients who can derive the greatest OS benefit from avelumab 1 L maintenance treatment.

## 2 MATERIALS AND METHODS

### 2.1 Patients and treatment

The design of the JAVELIN Bladder 100 trial (NCT02603432) has been described previously.^7^ Briefly, JAVELIN Bladder 100 is an international, multicenter, open‐label, randomized, phase 3 trial. Eligible patients had histologically confirmed unresectable aUC; no disease progression with 4 to 6 cycles of gemcitabine + cisplatin and/or gemcitabine + carboplatin prior to enrollment; and an ECOG PS of 0 or 1. After an interval of 4 to 10 weeks from last dose of chemotherapy, 700 patients were randomized (1:1) to receive either avelumab + BSC (*n* = 350) or BSC alone (*n* = 350). Randomization was stratified by metastatic site when chemotherapy was initiated (visceral vs. nonvisceral) and best response to 1 L chemotherapy (complete or partial response vs. stable disease). The primary endpoint was OS. The data cutoff date for OS follow‐up in this analysis was October 21, 2019, when median follow‐up for OS was >19 months in both study arms. As reported previously, the JAVELIN Bladder 100 trial was conducted in accordance with the ethics principles of the Declaration of Helsinki and Good Clinical Practice guidelines, defined by the International Council for Harmonisation. All patients provided written informed consent. The protocol, amendments, and informed consent forms were approved by the institutional review board or independent ethics committee at each trial site.^7^

### 2.2 Statistical analysis

#### 2.2.1 Machine learning analysis

Machine learning can expedite the identification of prognostic and predictive factors, particularly when a large set of baseline covariates is available. In our analysis, the dependent covariate was OS, which is the standard in oncology but is typically not addressed by existing machine learning pipelines due to a lack of support for censored outcome variables; therefore, we built a custom machine learning pipeline described in detail below. A range of baseline covariates from previously published and other sources were analyzed; assessment methods have been reported previously.^7^, ^8^, ^9^, ^16^, ^23^, ^24^

Covariates analyzed included demographic variables (age, sex, race, geographic region, ethnicity, smoking status, and body mass index); tumor/disease variables (ECOG PS, months since primary diagnosis, TNM stage at initial diagnosis and study entry, presence of measurable disease, sum of longest target lesion diameters, site of metastasis: visceral [lung or liver] vs. nonvisceral [including bone], and number of target and non‐target lesions); variables related to 1 L platinum‐based chemotherapy (regimen, response); hematology laboratory values (hemoglobin, platelets, lymphocytes, neutrophils, leukocytes, monocytes, eosinophils, basophils, neutrophil/leukocyte ratio, and systemic inflammation index); and chemistry laboratory values (alanine aminotransferase, albumin, alkaline phosphatase [ALP], serum amylase, aspartate aminotransferase, bilirubin, lactate dehydrogenase, C‐reactive protein [CRP], creatine kinase, creatinine, and gamma glutamyl transferase).^7^ Other covariates analyzed included patient‐reported outcomes (EQ‐5D‐5L, National Comprehensive Cancer Network/Functional Assessment of Cancer Therapy‐Bladder Symptom Index‐18 [NCCN FACT FBISI‐18])^9^; molecular or genetic biomarkers (PD‐L1, TMB, JAVELIN Renal 101 immune gene signature,^16^ and alleles encoding high‐affinity Fc gamma receptors [*FCGR2A/FGFR3A*])^23^; and cellular or pathologic characteristics, including several characteristics not analyzed in previous publications (tumor CD8+ T‐cell infiltration [center or margin], conventional immune phenotype in the tumor microenvironment [desert, excluded, or inflamed],^25^ lymphocyte density in tumor cell area, lymphocyte proportion in intratumoral stroma, and cell density [macrophages, fibroblasts, and granulocytes] in the tumor microenvironment).

**FIGURE 1:**
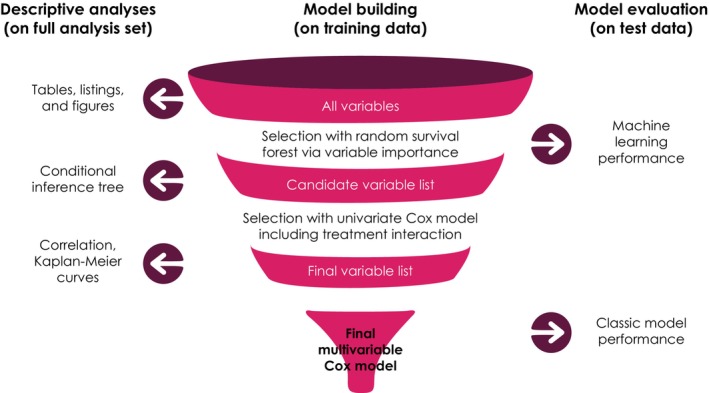
Analysis plan outline.

**FIGURE 2:**
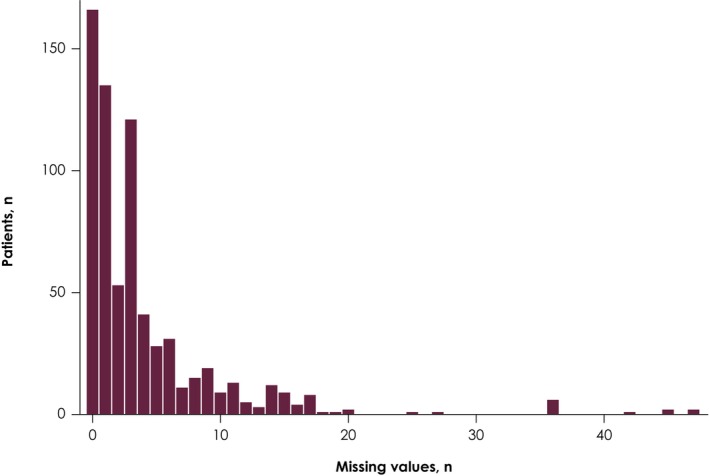
Missing data imputation. Factor variables were imputed with mode and numeric variables with median.

The dataset was split into training and test datasets and randomly allocated in a 5:1 ratio, respectively. Patients were stratified according to best response to 1 L chemotherapy (complete or partial response vs. stable disease), metastatic site when 1 L chemotherapy was initiated (visceral vs. nonvisceral), and receipt of subsequent therapy (yes vs. no). The training dataset was used for all model‐building decisions and the test dataset for final evaluation only (Figure 1). Details of missing data are shown in Figure 2; factor variables were explicitly coded (for frequently missing values) or were imputed (rare missing values imputed with mode and numeric variables imputed with median).

**FIGURE 3:**
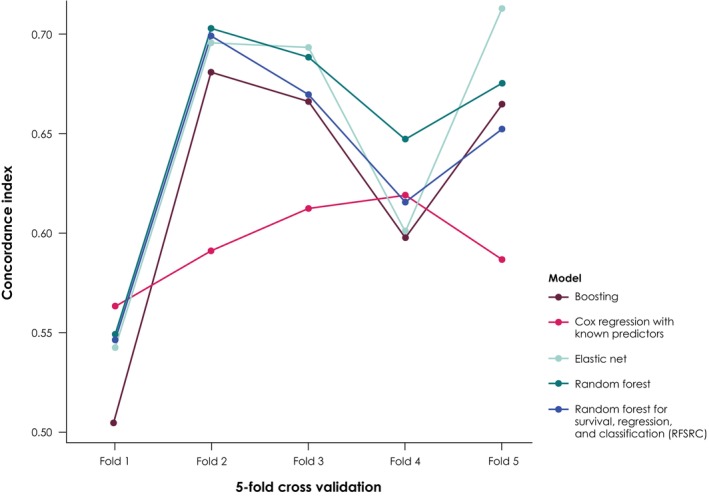
Benchmarking analysis of random survival forest versus other models. A five‐fold cross validation was performed, meaning that the training data were split into five equal parts; four parts were used for model development and one part for model evaluation. Random forest model was based on Liaw et al.^29^ Random forest for survival, regression, and classification (RFSRC) was based on Ishwaran et al.^30^

The predictive model was built using a random survival forest, which allows covariate selection of potentially high‐dimensional interactions.^26^ Random forests aggregate decision trees as base learners, perform well on large sets of covariates, and are robust even if assumptions are violated. The model fit was performed on pooled data from both treatment arms and on treatment arms independently. Variables were selected if they were deemed important by at least two of the three models using permutation‐based variable importance (vimp) measures, with threshold = mean (vimp) + SD (vimp).^27^ Sensitivity analyses were performed using different sets of covariates. The random survival forest was benchmarked to ensure that its performance was competitive versus various other machine learning algorithms, including elastic net and boosting (Figure 3).^28^, ^29^, ^30^ In addition, univariate Cox modeling was performed for each variable with treatment interaction, allowing differentiation between prognostic and predictive effects.

All analyses were performed using R software version 4.1.1. The main random forest analysis used the randomForestSRC package, and benchmarking with additional machine learning models used the tidymodels set of packages, including the censored package.

#### 2.2.2 Interpretation and Cox modeling

All candidate covariates from the data‐driven variable selection were examined using descriptive analyses, Spearman correlation, and Kaplan–Meier analysis of OS. For numeric variables, subgroups for Kaplan–Meier analyses of OS were created using data‐driven cutoffs obtained from conditional inference tree models.^31^ After the final selection of prognostic and predictive baseline factors, a multivariate Cox model was fitted that allowed clinical insights and interpretation. The set of variables selected in machine learning modeling was augmented with known prognostic/predictive factors. Final variable selection was performed using stepwise model selection using the Akaike information criterion on the training dataset. The final Cox model was evaluated on the test dataset, and its performance was compared with the performance of the random survival forest. Harrell's C‐index (concordance) was used as an evaluation metric.

## 3 RESULTS

### 3.1 Machine learning analysis

**FIGURE 4:**
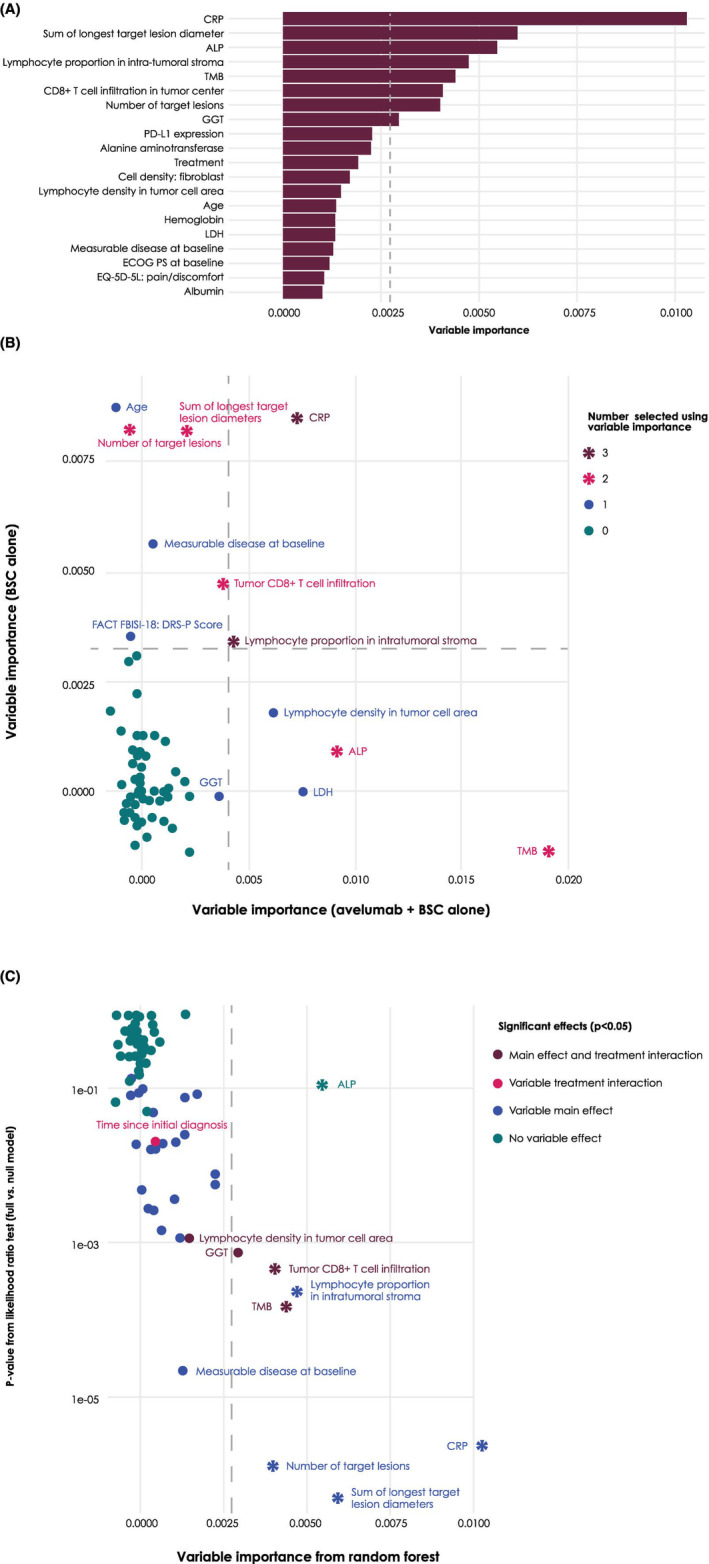
Variable selection using machine learning analysis. (A) Variable importance from the random survival forest (pooled analysis across treatment arms). The dashed line represents the threshold mean (variable importance) + SD (variable importance). Variables were sorted by importance, and the top 20 variables are displayed. (B) Variable importance by treatment arm from the random forest model. Color‐coding corresponds to the number of analyses in which the specific variable was selected, that is, variable importance (x) > mean (variable importance) + SD (variable importance). (C) Variable importance from pooled analysis versus likelihood ratio test from univariate Cox modeling. The *p* value is derived from comparing the full model to the model with a treatment main effect only. AIC, Akaike information criterion; ALP, alkaline phosphatase; BSC, best supportive care; CRP, C‐reactive protein; ECOG PS, Eastern Cooperative Oncology Group performance status; GGT, gamma glutamyl transferase; LDH, lactate dehydrogenase; PD‐L1, programmed death ligand 1; TMB, tumor mutational burden.

Results from the primary random survival forest analysis determining the relative importance of all baseline variables, considered in terms of their association with OS (in the pooled analysis across treatment arms), and the ranking of the most important variables are shown in Figure 4A. Variables selected in the pooled model as well as in both the avelumab + BSC and BSC alone arm were CRP level and lymphocyte proportion in intratumoral stroma (Figure 4B). Variables selected in the pooled model and the avelumab + BSC arm were TMB and ALP level. Variables selected in the pooled model and the BSC arm alone were sum of longest target lesion diameters, number of target lesions, and CD8+ T‐cell infiltration in the tumor center. A summary of sensitivity analyses using a subset of covariate groups is shown in Table 1. These revealed additional candidates with potential prognostic or predictive value, including ECOG PS, time since initial diagnosis, PD‐L1 expression, and age.

**TABLE 1:** Summary of sensitivity analyses.

Candidate variables	Selected variables	Out‐of‐bag error*
All covariates	Sum of longest target lesion diameters, number of target lesions, CRP, ALP, TMB, lymphocyte proportion in intratumoral stroma, and tumor CD8+ T‐cell infiltration	0.3736
Clinical covariates	Age and number of target lesions	0.3976
Clinical and laboratory covariates	PD‐L1, sum of longest target lesion diameters, number of target lesions, CRP, and ALP	0.3827
Clinical covariates and biomarkers	ECOG PS at baseline, sum of longest target lesion diameters, number of target lesions, TMB, and lymphocyte proportion in intratumoral stroma	0.3843
Clinical covariates and tumor characteristics	ECOG PS at baseline, time since initial diagnosis, sum of longest target lesion diameters, and number of target lesions	0.4076

Abbreviations: ALP, Alkaline phosphatase; CRP, C‐reactive protein; ECOG PS, Eastern Cooperative Oncology Group performance status; PD‐L1, programmed death ligand 1; TMB, tumor mutational burden.

* Out‐of‐bag prediction error is calculated by evaluating the performance on those observations that were not used in building the base‐learner tree.

All covariates were screened using univariate Cox regression models predicting OS and including treatment interaction effects (Figure 4C). Both a main effect and a treatment interaction were seen for TMB, CD8+ T‐cell infiltration in the tumor center, lymphocyte density in the tumor cell area, and gamma glutamyl transferase; however, the last two variables were not strongly associated with OS and were not selected in the random survival forest analyses. Strong main effects were seen for sum of longest target lesion diameters, number of target lesions, CRP level, lymphocyte proportion in intratumoral stroma, and presence of measurable disease. All variables except for presence of measurable disease were selected in random survival forest analyses (measurable disease was strongly correlated with other candidate covariates).

### 3.2 Final Cox model

The list of candidates selected by random survival forest included sum of longest target lesion diameters, number of target lesions, ALP level, CRP level, lymphocyte proportion in intratumoral stroma, CD8+ T‐cell infiltration in the tumor center, and TMB. Additional candidates selected in sensitivity analyses, each using a different subset of covariate groups, included time since initial diagnosis, ECOG PS at baseline, and PD‐L1 status. The candidate variables were augmented with known predictors: treatment arm, age, and visceral versus nonvisceral metastases. Correlations between all covariates identified were assessed (Figure S1). Factors not included in the final list because of strong correlations with sum of longest diameters were number of target lesions and presence of measurable disease. Kaplan–Meier analyses of OS in subgroups defined by various covariates are shown in Figure S2.

The summary of final model building using the training dataset is shown in Table 2. After data‐driven, stepwise model selection, the final Cox model for OS was obtained (Table 3). In the overall population, the most relevant prognostic variables for OS (*p* < 0.1) were sum of longest target lesion diameters, CRP level, ALP level, and lymphocyte proportion in the intratumoral stroma. However, the magnitude of the treatment effect can only be interpreted correctly in the context of its respective interaction effects.

**TABLE 2:** Final modeling on training dataset.

Model	Variables	*n*	No. of events	Concordance	Standard error/concordance	AIC
Null model	Treatment	559	257	0.554	0.017	2939
Known predictors	Treatment, age, PD‐L1, ECOG PS at baseline, site of metastasis	559	257	0.611	0.019	2921
Selected candidates from machine learning analysis	Sum of longest target lesion diameters, CRP, ALP, lymphocyte proportion in intratumoral stroma, TMB, tumor CD8+ T‐cell infiltration, ECOG PS at baseline, time since initial diagnosis, PD‐L1, and age	559	257	0.658	0.017	2872
Full model with selected candidates and known predictors	Treatment, age, PD‐L1, ECOG PS at baseline, site of metastasis, sum of longest target lesion diameters, CRP, ALP, lymphocyte proportion in intratumoral stroma, TMB, tumor CD8+ T‐cell infiltration, and time since initial diagnosis	559	257	0.670	0.017	2867
Final model	Treatment, ECOG PS at baseline, site of metastasis*treatment, sum of longest target lesion diameters, CRP, ALP, lymphocyte proportion in intratumoral stroma, TMB*treatment, and tumor CD8+ T‐cell infiltration*treatment	559	257	0.678	0.017	2855

*Note*: Asterisks indicate that both a main effect and an interaction effect between the respective variables were included.

Abbreviations: AIC, Akaike information criterion; ALP, alkaline phosphatase; CRP, C‐reactive protein; ECOG PS, Eastern Cooperative Oncology Group performance status; PD‐L1, programmed death ligand 1; TMB, tumor mutational burden.

**TABLE 3:** Proportional hazards regression model for OS after variable selection.

Predictor	Level	HR (95% CI)	*p* value
Treatment	BSC versus avelumab + BSC	1.628 (0.59–4.52)	0.3503
ECOG PS at baseline	0 versus ≥1	1.249 (0.95–1.64)	0.1112
Site of metastases	Nonvisceral versus visceral	0.856 (0.59–1.23)	0.4049
Sum of longest target lesion diameters		1.012 (1.01–1.02)	<0.0001
CRP		1.024 (1.02–1.03)	<0.0001
ALP		1.093 (1.01–1.18)	0.0212
Lymphocyte proportion in intratumoral stroma		0.973 (0.95–1.00)	0.0398
TMB		0.973 (0.92–1.03)	0.3111
Tumor CD8+ T‐cell infiltration		0.987 (0.92–1.06)	0.7091
Treatment: site of metastases	Avelumab + BSC: visceral	1.795 (1.03–3.14)	0.0402
Treatment: TMB	Avelumab + BSC	0.906 (0.83–0.98)	0.0196
Treatment: tumor CD8+ T‐cell infiltration	Avelumab + BSC	0.871 (0.74–1.02)	0.0894

*Note*: Converge.Concordance = 0.697, standard error = 0.018. Likelihood ratio test = 122.76 on 12 degrees of freedom, *p* < 0.05. Akaike information criterion = 2460.5, *n* = 512 (observations without missing values on any of the predictors), number of events = 225.

Abbreviations: ALP, alkaline phosphatase; BSC, best supportive care; CI, confidence internal; CRP, C‐reactive protein; ECOG PS, Eastern Cooperative Oncology Group performance status; HR, hazard ratio; OS, overall survival; PD‐L1, programmed death ligand 1; TMB, tumor mutational burden.

A predictive effect was observed for TMB, CD8+ T‐cell infiltration in the tumor center, and visceral metastasis. The model for the subpopulation of patients with PD‐L1+ tumors showed similar trends (Table S1). The prediction performance (concordance) was 71.1% using test data for the random survival forest and 70.9% for the final Cox model. A summary of the final model with only main effects included is shown in Table S2.

## 4 DISCUSSION

The specific mechanism of action of immunotherapies creates multiple methodological challenges when assessing long‐term treatment benefit.^32^, ^33^ Challenges include assessing non‐proportional hazards, delayed separation of OS curves, and unobserved heterogeneity in the patient population. Challenges of assessing non‐proportional hazards and delayed separation of OS curves have been discussed extensively in the published literature.^34^, ^35^, ^36^ This analysis focused on the challenge of capturing the heterogeneity in treatment effect and OS by identifying characteristics at baseline that may have prognostic or predictive effects on OS using machine learning analyses.

Machine learning has been used previously to identify prognostic factors in various cancers.^20^, ^37^, ^38^, ^39^ In this study, machine learning was used to identify baseline factors associated with OS in patients with aUC who had received 1 L platinum‐based chemotherapy without disease progression who were treated with avelumab + BSC or BSC alone in the JAVELIN Bladder 100 trial.^7^, ^8^ All analyses were exploratory and were performed for hypothesis‐generating purposes only. Although previous analyses from JAVELIN Bladder 100 have assessed associations between various factors and OS in separate analyses,^7^, ^23^, ^40^ no comprehensive analysis incorporating a wide range of baseline factors and adjusting for their respective effects has been reported to date. Our analyses successfully selected factors independently associated with OS, confirming the importance of some previously reported factors. Additionally, novel cellular or pathologic characteristics in the JAVELIN Bladder 100 dataset were analyzed for the first time, including conventional immune phenotype in the tumor microenvironment, lymphocyte density in the tumor cell area or lymphocyte proportion in intratumoral stroma, and density of various cell types in the tumor microenvironment. Overall, our studies further support the use of a machine learning approach to identify potential prognostic and predictive factors in clinical trial populations.

Longer OS irrespective of treatment was independently associated with lower sum of longest target lesion diameters, lower CRP and ALP levels, and higher lymphocyte proportion in intratumoral stroma (i.e., prognostic factors). The sum of longest target lesion diameters is a measure of tumor burden and has been identified as a prognostic factor in various tumor types, including UC.^41^, ^42^, ^43^, ^44^ CRP is a systemic inflammation and is an established predictive marker in urological cancers.^45^ CRP was previously concluded to be a predictive marker in a previous analysis of JAVELIN Bladder 100^40^ and for 1 L avelumab + axitinib response in patients with advanced renal cell carcinoma in JAVELIN Renal 101.^46^ Additionally, ALP has been identified previously as a poor prognostic marker in patients with aUC,^47^, ^48^ and tumor‐infiltrating lymphocyte density has been associated with patient outcomes in various cancers, including UC.^49^, ^50^ Overall, our analyses suggest that these factors are specifically relevant in the 1 L maintenance setting in aUC.

In addition, longer OS with avelumab 1 L maintenance treatment was associated with a high TMB, a higher level of CD8+ T‐cell infiltration in the tumor center, and absence of visceral metastases (i.e., predictive factors). TMB is a widely recognized biomarker indicating increased sensitivity to ICI treatment in several tumors, including aUC, although thresholds used have varied between studies.^18^, ^51^, ^52^ In the US, pembrolizumab monotherapy has been approved for the treatment of patients with advanced solid tumors with high TMB (≥10 mutations/megabase) based on results from KEYNOTE‐158, a multicohort, open‐label, non‐randomized, phase 2 study.^53^, ^54^ High CD8+ T‐cell density in tumors, or high levels of tumor‐infiltrating lymphocytes in general, are indicators of preexisting immune activation.^55^ Both high TMB and high CD8+ T‐cell infiltration were associated with longer OS with avelumab 1 L maintenance treatment for aUC in a previous analysis.^23^ Additionally, high TMB and intratumoral CD8+ T‐cell density in the invasive margin have been associated with better outcomes in patients receiving avelumab treatment for metastatic MCC.^56^, ^57^ Lastly, visceral metastases are an established risk factor for shorter OS in patients with aUC.^14^

Overall, data from the JAVELIN Bladder 100 trial reported previously, along with real‐world studies in heterogeneous populations, have provided high‐level evidence to support the use of avelumab 1 L maintenance as a standard of care for eligible patients with aUC with different characteristics, as recommended in international treatment guidelines.^10^, ^11^ Additional exploratory analyses from other studies of avelumab 1 L maintenance and prospective studies are needed to further evaluate the clinical relevance of prognostic and predictive factors identified.

## AUTHOR CONTRIBUTIONS

**Juliane Manitz:** Conceptualization (equal); formal analysis (equal); methodology (equal); software (equal); writing – original draft (equal); writing – review and editing (equal). **Aslihan Gerhold‐Ay:** Conceptualization (equal); methodology (equal); validation (equal); writing – review and editing (equal). **Pascal Kieslich:** Conceptualization (equal); formal analysis (equal); methodology (equal); software (equal); writing – review and editing (equal). **Parantu Shah:** Conceptualization (equal); writing – review and editing (equal). **Thomas Mrowiec:** Conceptualization (equal); writing – review and editing (equal). **Karin Tyroller:** Conceptualization (equal); methodology (equal); supervision (equal); validation (equal); writing – review and editing (equal).

## FUNDING INFORMATION

The JAVELIN Bladder 100 trial was sponsored by Pfizer and was previously conducted under an alliance between the healthcare business of Merck KGaA, Darmstadt, Germany (CrossRef Funder ID: 10.13039/100009945) and Pfizer. The analyses reported in this manuscript were funded by the healthcare business of Merck KGaA, Darmstadt, Germany.

## CONFLICT OF INTEREST STATEMENT

JM, PS, and KT are employees of EMD Serono; AGA, PK, and TM are employees of the healthcare business of Merck KGaA, Darmstadt, Germany.

## ETHICS STATEMENT

Not applicable for the analyses reported.

## Supporting information


Data S1:

## Data Availability

Any requests for data by qualified scientific and medical researchers for legitimate research purposes will be subject to the healthcare business of Merck KGaA, Darmstadt, Germany's (CrossRef Funder ID: 10.13039/100009945) Data Sharing Policy. All requests should be submitted in writing to the healthcare business of Merck KGaA, Darmstadt, Germany's data sharing portal (https://www.emdgroup.com/en/research/our‐approach‐to‐research‐and‐development/healthcare/clinical‐trials/commitment‐responsible‐data‐sharing.html). When the healthcare business of Merck KGaA, Darmstadt, Germany has a co‐research, co‐development, or co‐marketing or co‐promotion agreement, or when the product has been out‐licensed, the responsibility for disclosure might be dependent on the agreement between parties. Under these circumstances, the healthcare business of Merck KGaA, Darmstadt, Germany will endeavor to gain agreement to share data in response to requests.
