# Supplementary material for: Avelumab first‐line maintenance in advanced urothelial carcinoma: Complete screening for prognostic and predictive factors using machine learning in the JAVELIN Bladder 100 phase 3 trial
Source: Cancer Med. 2024 Jun 24;13(12):e7411. doi: 10.1002/cam4.7411 (PMC11194683; doi:10.1002/cam4.7411)
Supplement: Supplementary file 1 — Data S1: [file CAM4-13-e7411-s001.docx]

**SUPPLEMENTARY TABLES**

**Supplementary Table 1. Proportional hazards regression model for OS in patients with PD-L1 positive tumors.** Converged. Concordance=0.718, standard error=0.022. Likelihood ratio test=71.3 on 12 degrees of freedom, p<0.05. Akaike information criterion=1187.84, n=303, number of events=119.

| **Predictor** | **Level** | **HR (95% CI)** | **P value** |
| --- | --- | --- | --- |
| Treatment | BSC vs avelumab + BSC | 1.255 (0.29-5.36) | 0.7589 |
| ECOG PS at baseline | 0 vs ≥1 | 1.064 (0.73-1.55) | 0.7439 |
| Site of metastases | Nonvisceral vs visceral | 0.872 (0.54-1.40) | 0.5724 |
| Sum of longest target lesion diameters |  | 1.011 (1.00-1.02) | 0.0031 |
| CRP |  | 1.021 (1.01-1.03) | 0.0001 |
| ALP |  | 1.143 (1.03-1.27) | 0.0099 |
| Lymphocyte proportion in intratumoral stroma |  | 0.961 (0.93-1.00) | 0.0297 |
| TMB |  | 0.936 (0.87-1.01) | 0.0946 |
| CD8+ T-cell infiltration |  | 1.007 (0.94-1.08) | 0.8560 |
| Treatment: site of metastases | Avelumab + BSC: visceral | 1.495 (0.70-3.17) | 0.2951 |
| Treatment: TMB | Avelumab + BSC | 0.941 (0.84-1.06) | 0.3188 |
| Treatment: tumor CD8+ T-cell infiltration | Avelumab + BSC | 0.865 (0.71-1.05) | 0.1371 |

**ALP**, alkaline phosphatase; **BSC**, best supportive care; **CI**, confidence interval; **CRP**, C-reactive protein; **ECOG PS**, Eastern Cooperative Oncology Group performance status; **HR,** hazard ratio; **OS**, overall survival; **PD-L1**, programmed death ligand 1; **TMB**, tumor mutational burden.

**Supplementary Table 2. Proportional hazards regression model for OS with only main effects.** Converged. Concordance=0.683, standard error=0.018. Likelihood ratio test=108.28 on 9 degrees of freedom, p<0.05. Akaike information criterion=2468.97, n=512, number of events=225.

| **Predictor** | **Level** | **HR (95% CI)** | **P value** |
| --- | --- | --- | --- |
| Treatment | BSC vs avelumab + BSC | 0.660 (0.51-0.86) | 0.0021 |
| ECOG PS at baseline | 0 vs ≥1 | 1.223 (0.93-1.60) | 0.1466 |
| Site of metastases | Nonvisceral vs visceral | 1.145 (0.87-1.50) | 0.3289 |
| Sum of longest target lesion diameters |  | 1.012 (1.01-1.02) | <0.0001 |
| CRP |  | 1.023 (1.02-1.03) | <0.0001 |
| ALP |  | 1.086 (1.01-1.17) | 0.0305 |
| Lymphocyte proportion in intratumoral stroma |  | 0.970 (0.94-1.00) | 0.0250 |
| TMB |  | 0.927 (0.89-0.97) | 0.0003 |
| CD8+ T-cell infiltration |  | 0.952 (0.89-1.02) | 0.1549 |

**ALP**, alkaline phosphatase; **BSC**, best supportive care; **CRP**, C-reactive protein; **ECOG PS**, Eastern Cooperative Oncology Group performance status; **OS**, overall survival; **PD-L1**, programmed death ligand 1; **TMB**, tumor mutational burden.

**SUPPLEMENTARY FIGURES**

**Supplementary Figure 1. Correlations between candidate variables**

**
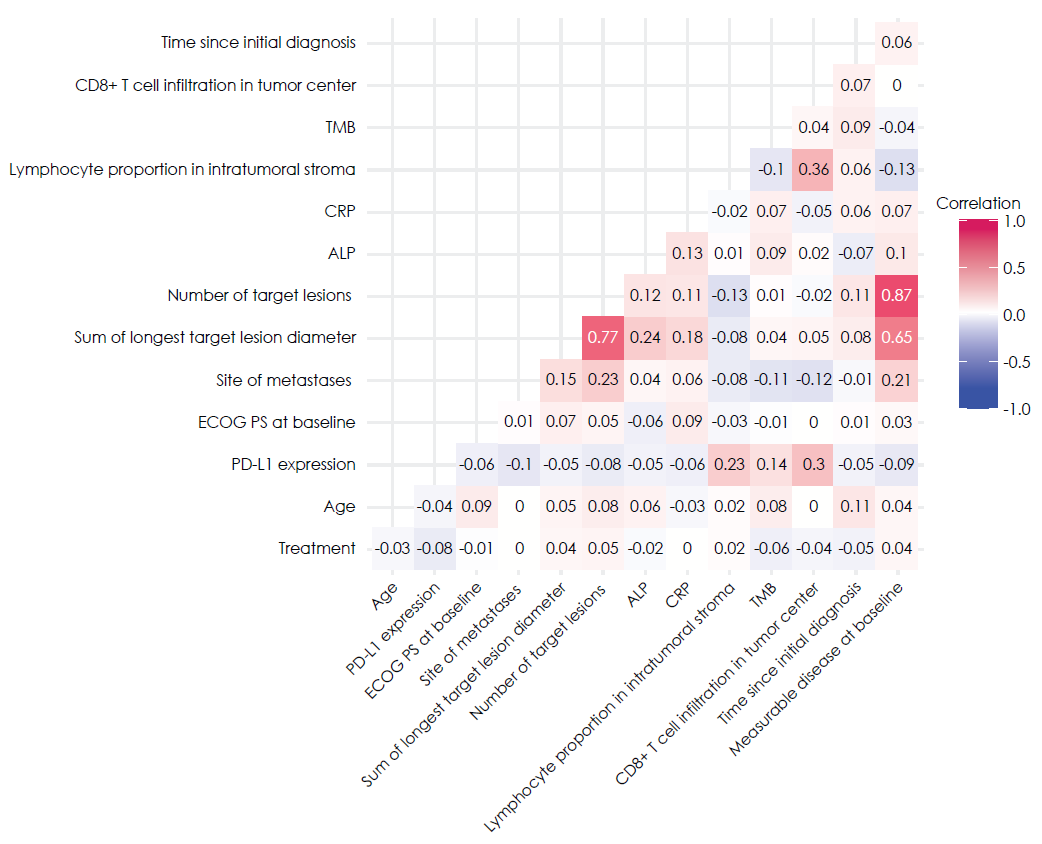
**

**ALP**, alkaline phosphatase; **CRP**, C-reactive protein; **TMB**, tumor mutational burden.

**Supplementary Figure 2. Kaplan-Meier analysis of overall survival by treatment arm in subgroups defined by covariates**. Only selected candidates are presented. The source of the presented cutoffs were derived from conditional inference trees.

1. **Sum of longest target lesion diameter** **
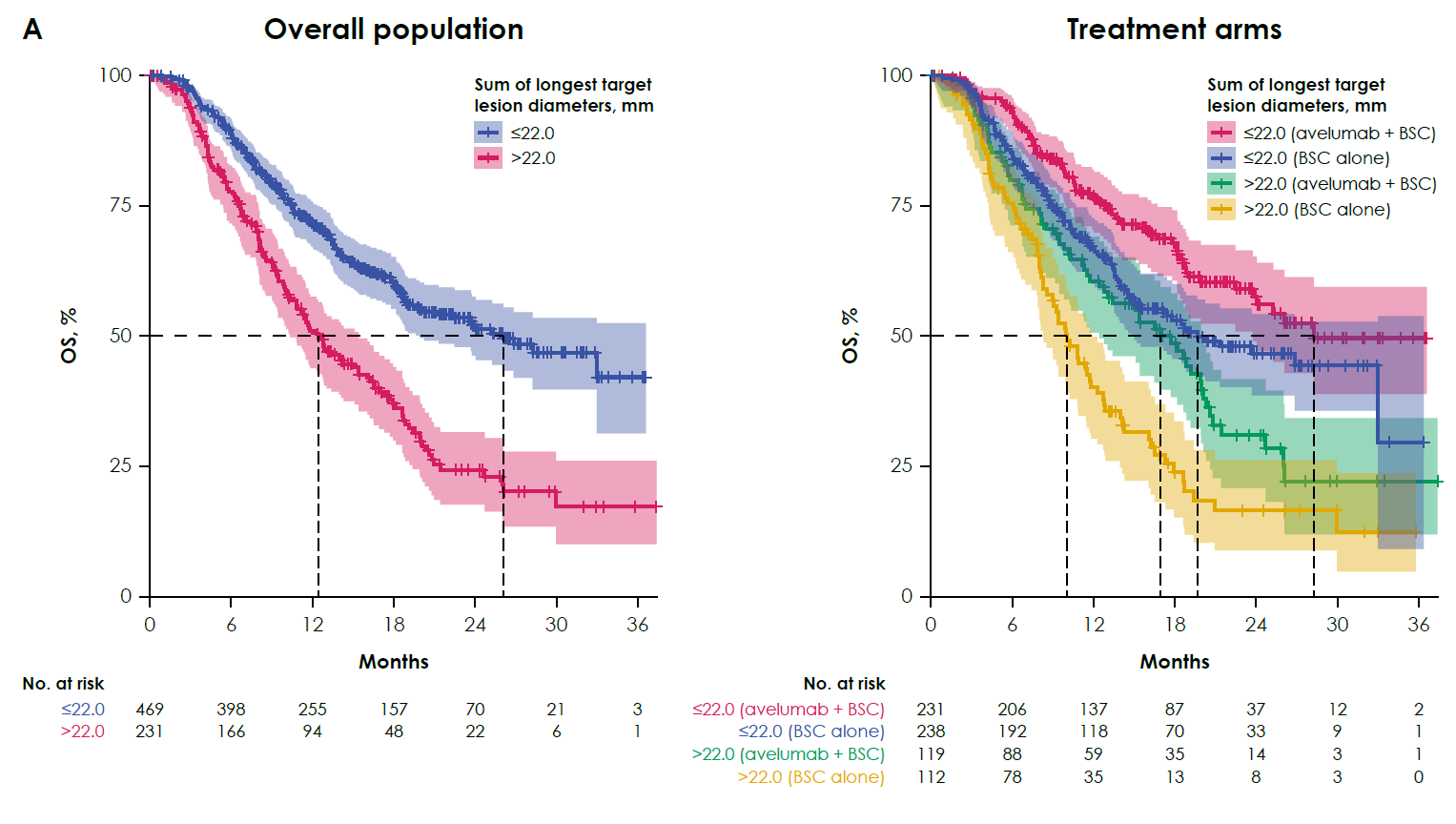
**
2. **CRP level**

**
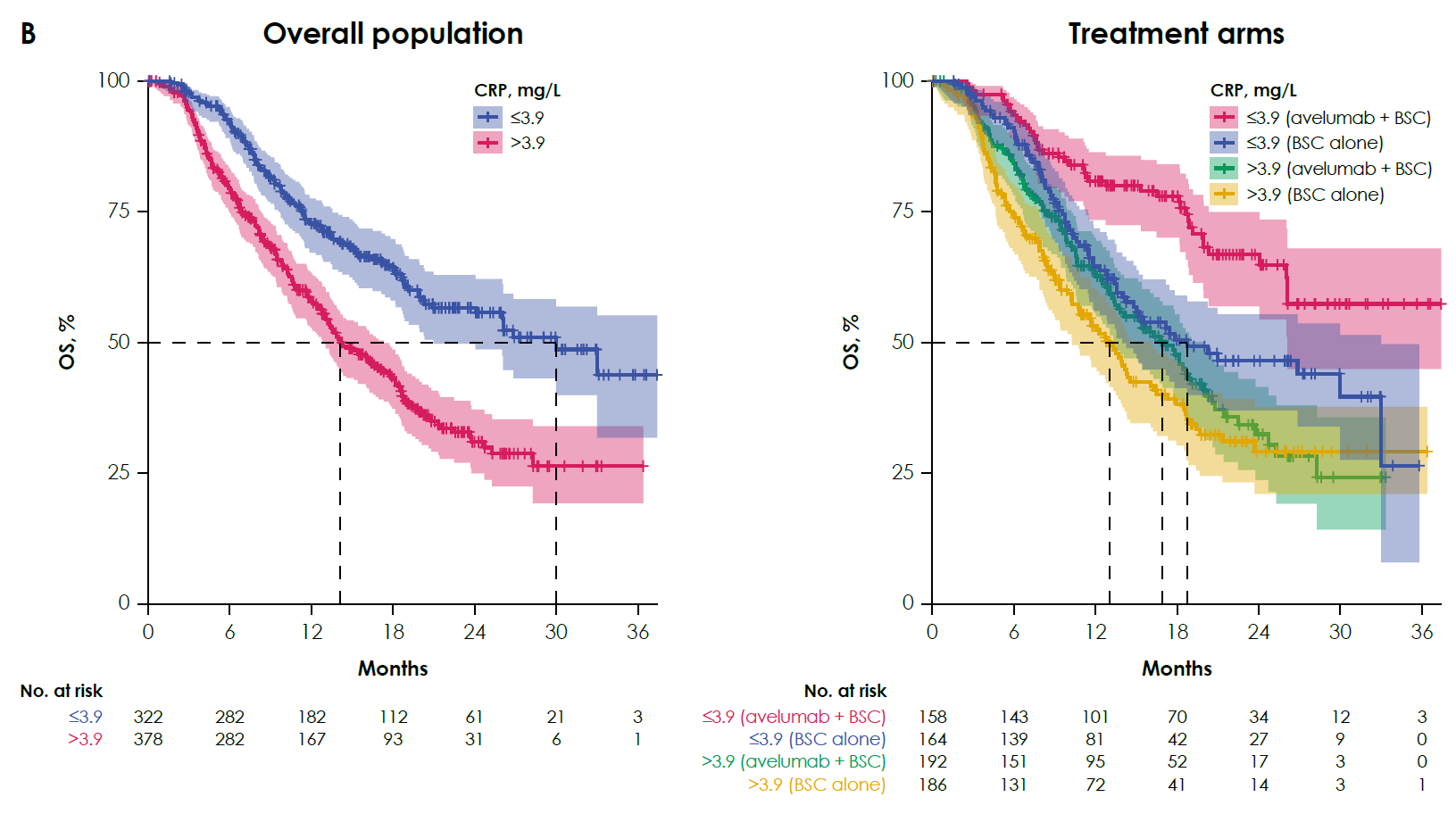
**

1. **TMB**

**
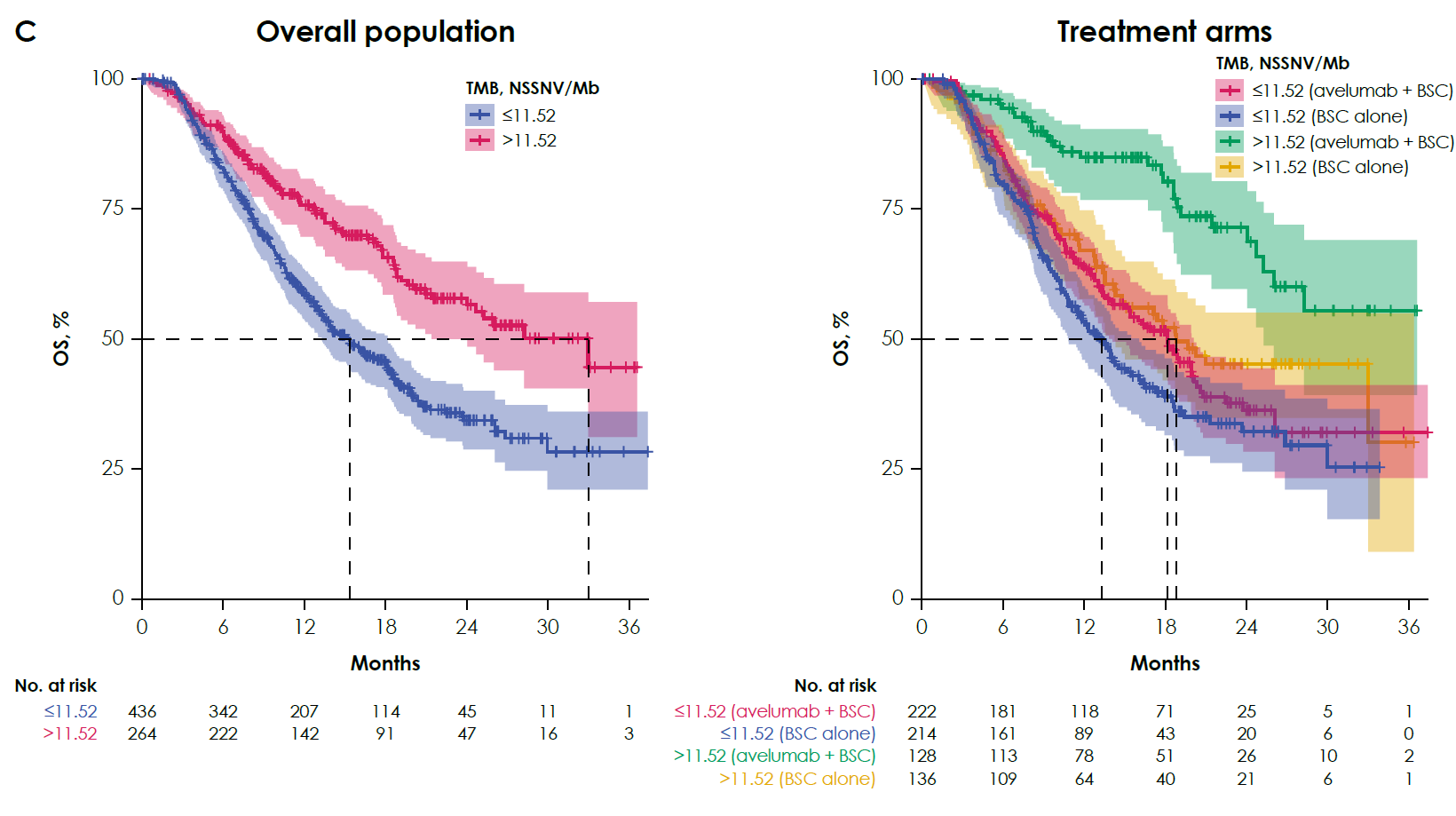
**

1. **ALP level**

**
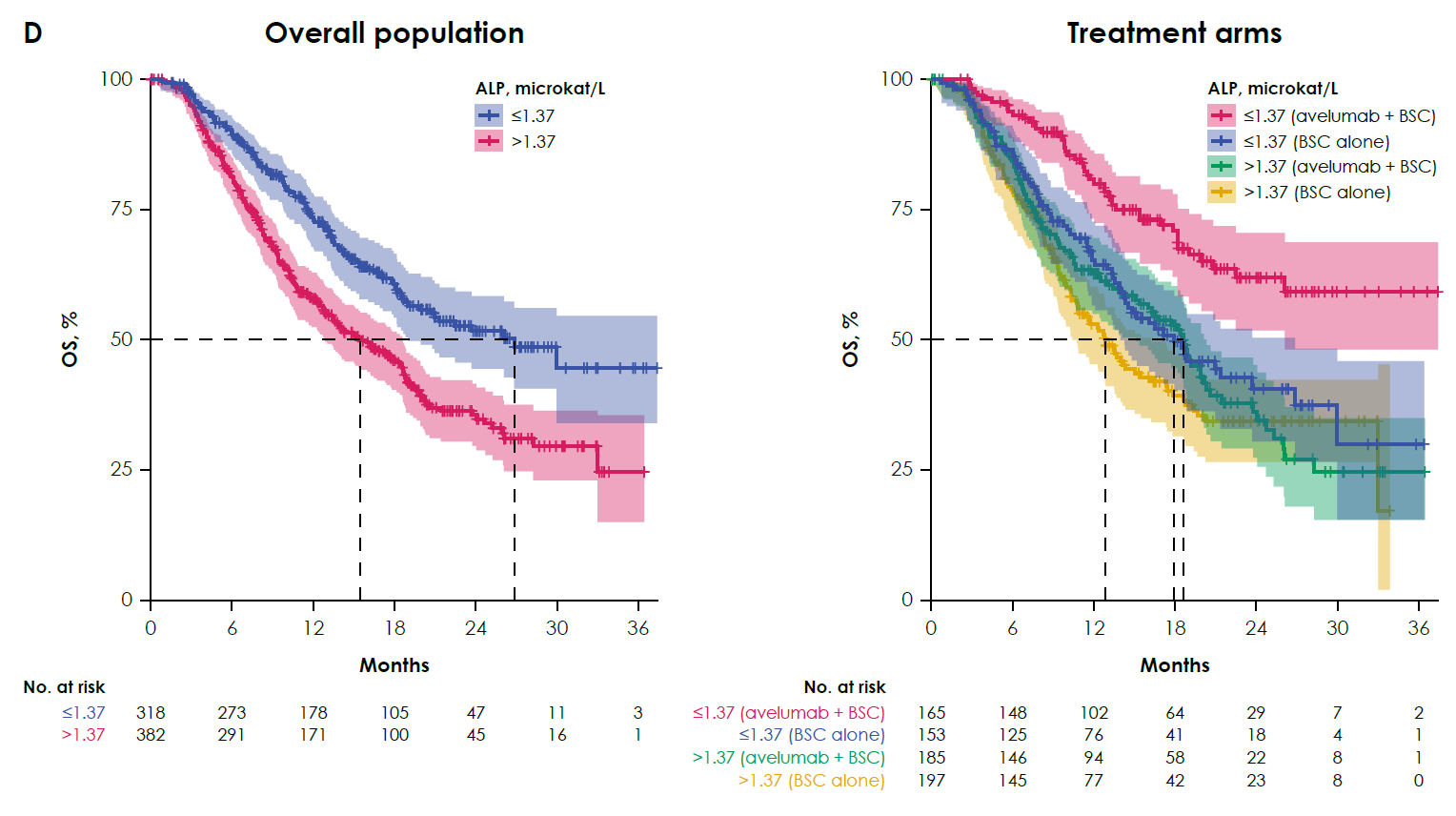
**

1. **Number of target lesions**

**
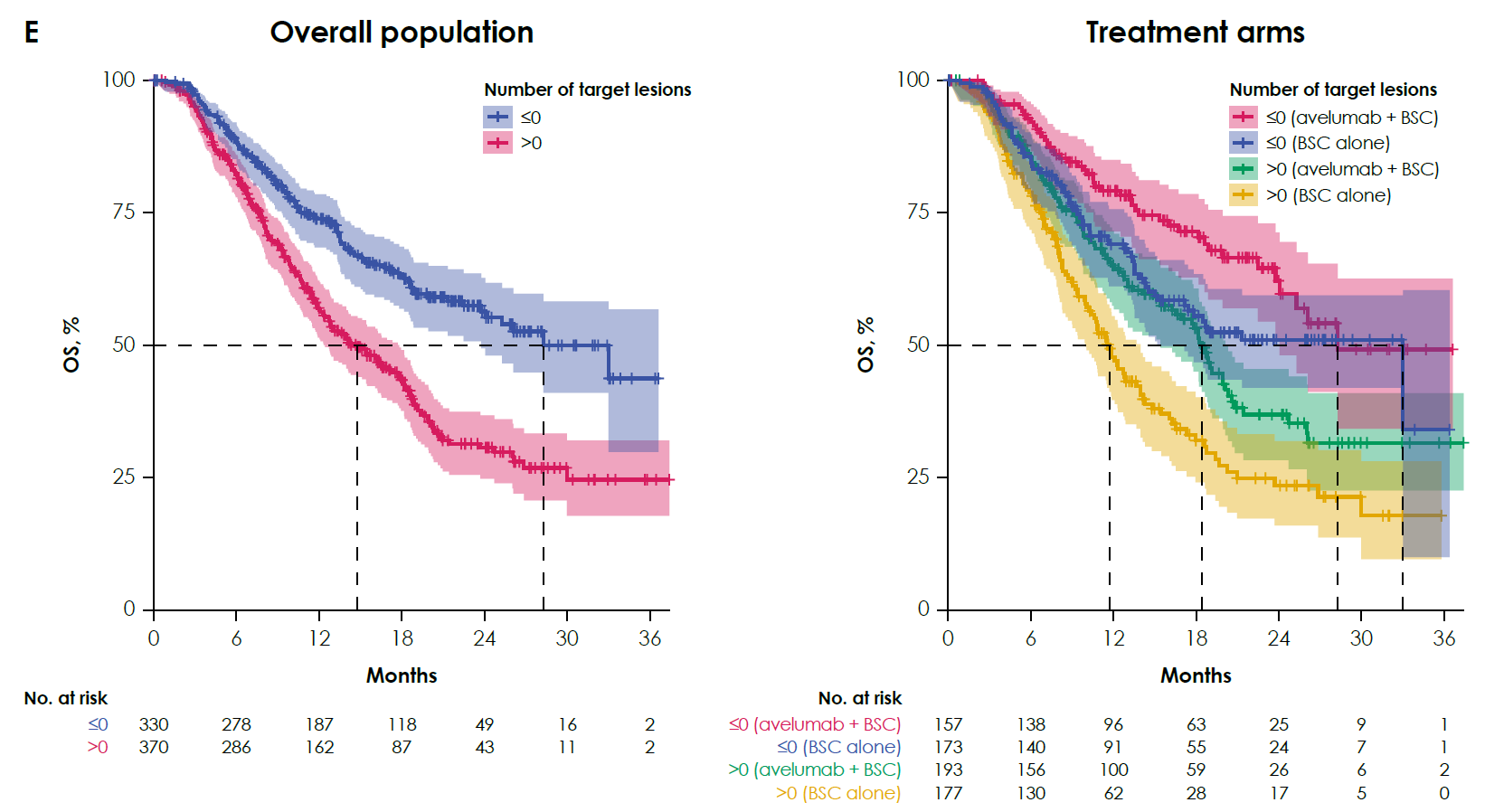
**

1. **Lymphocyte proportion in intratumoral stroma**

**
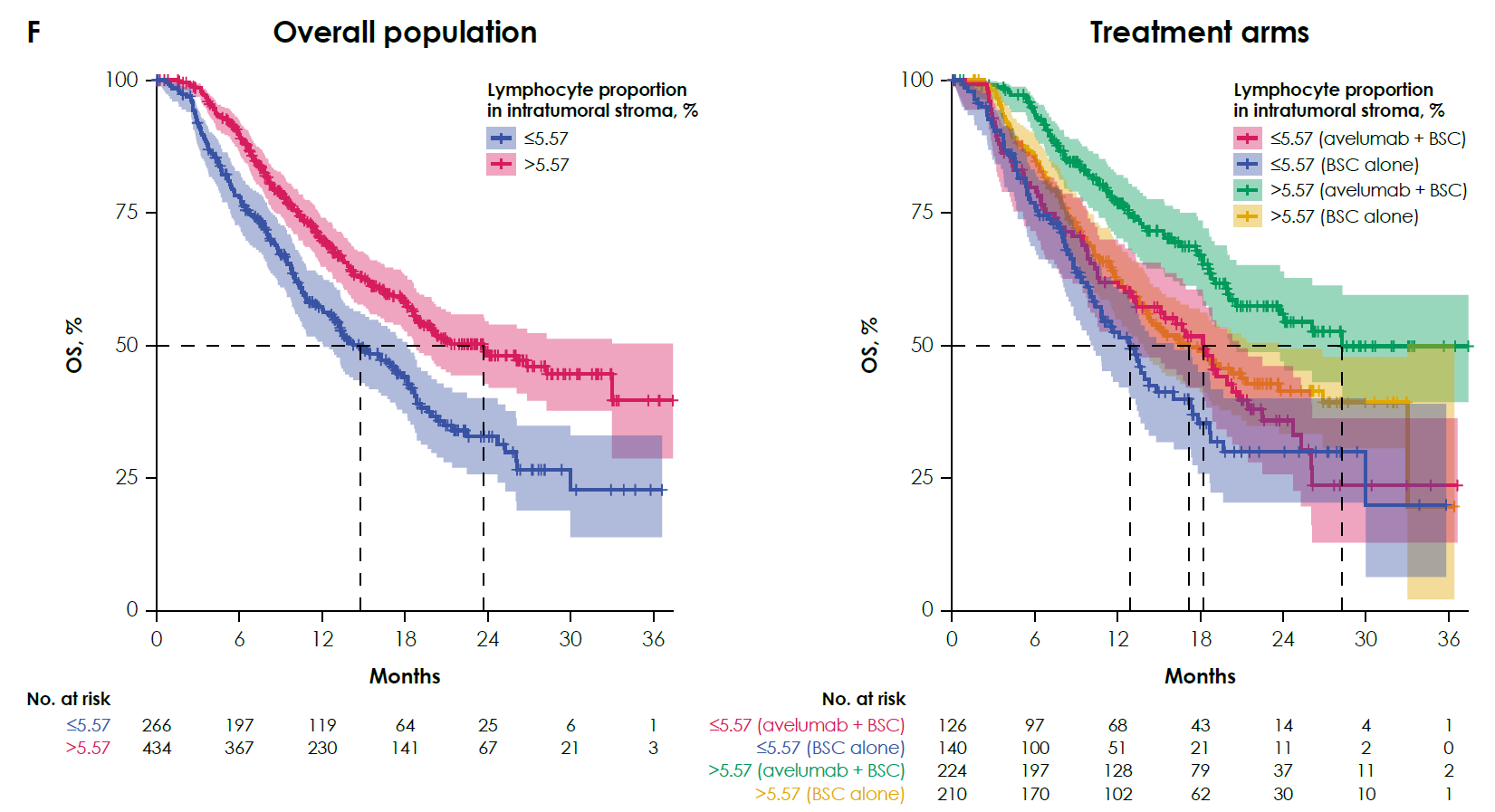
**

1. **CD8+ T cell infiltration in the tumor center**

**
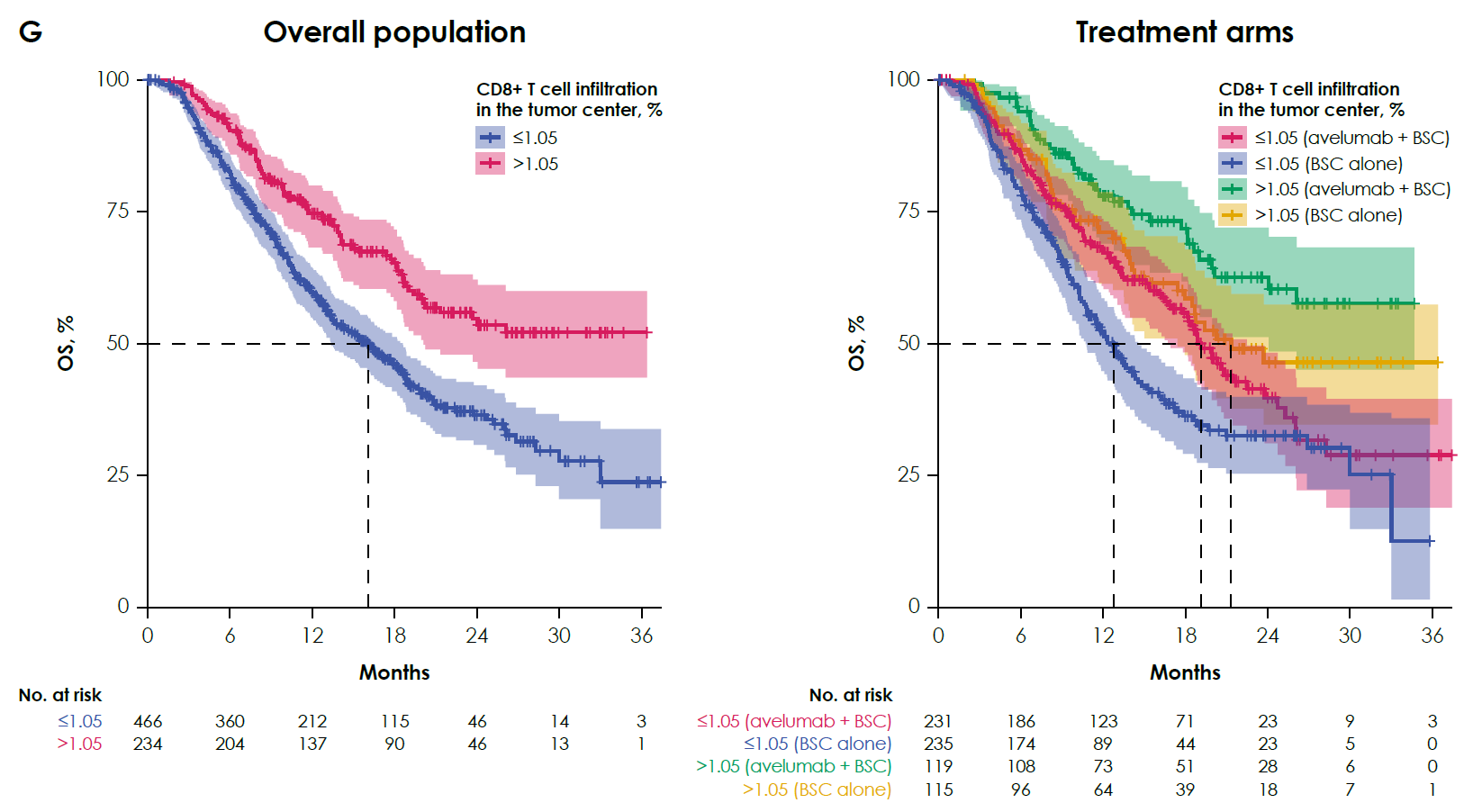
**

1. **ECOG PS**

**
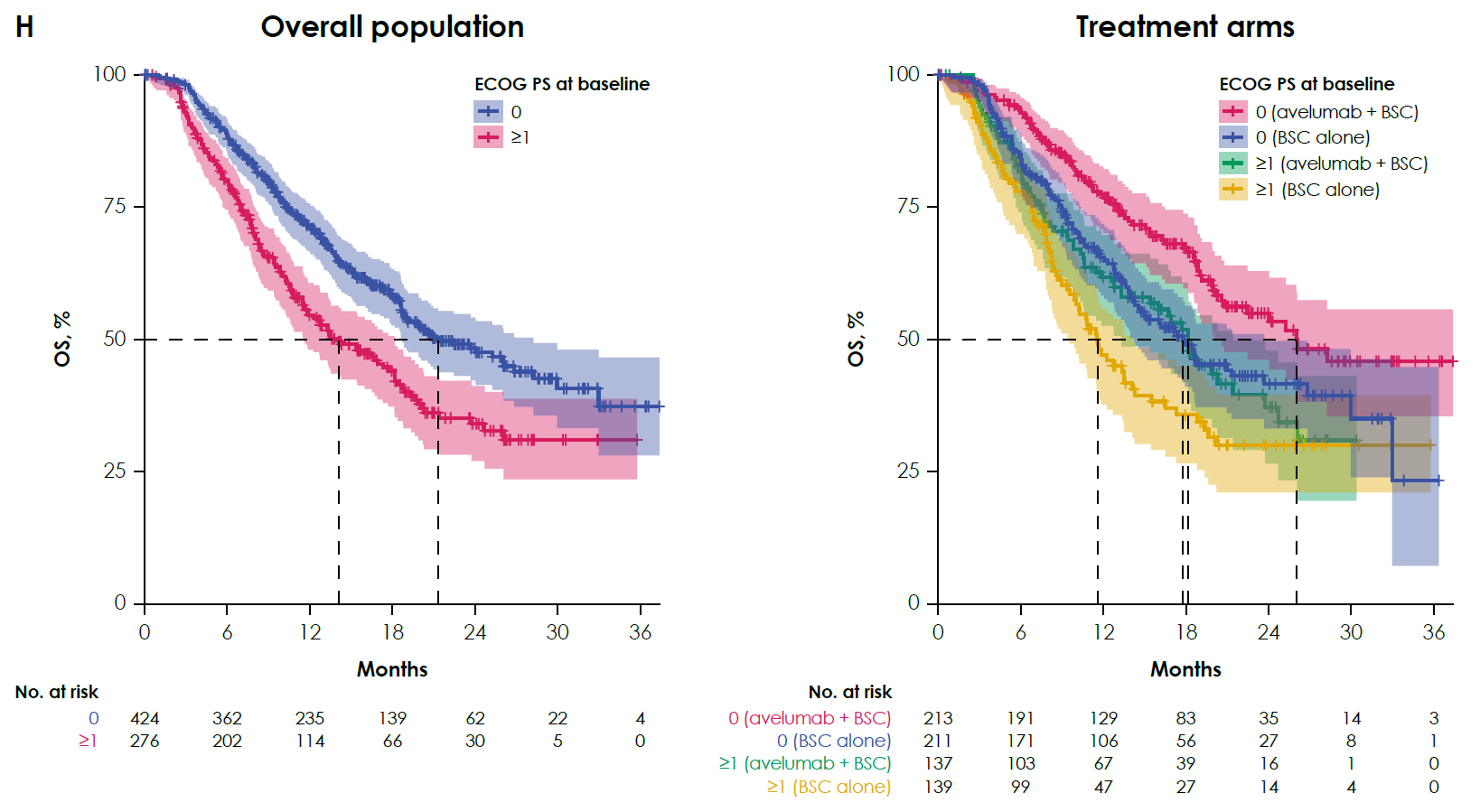
**

1. **Time since initial diagnosis**

**
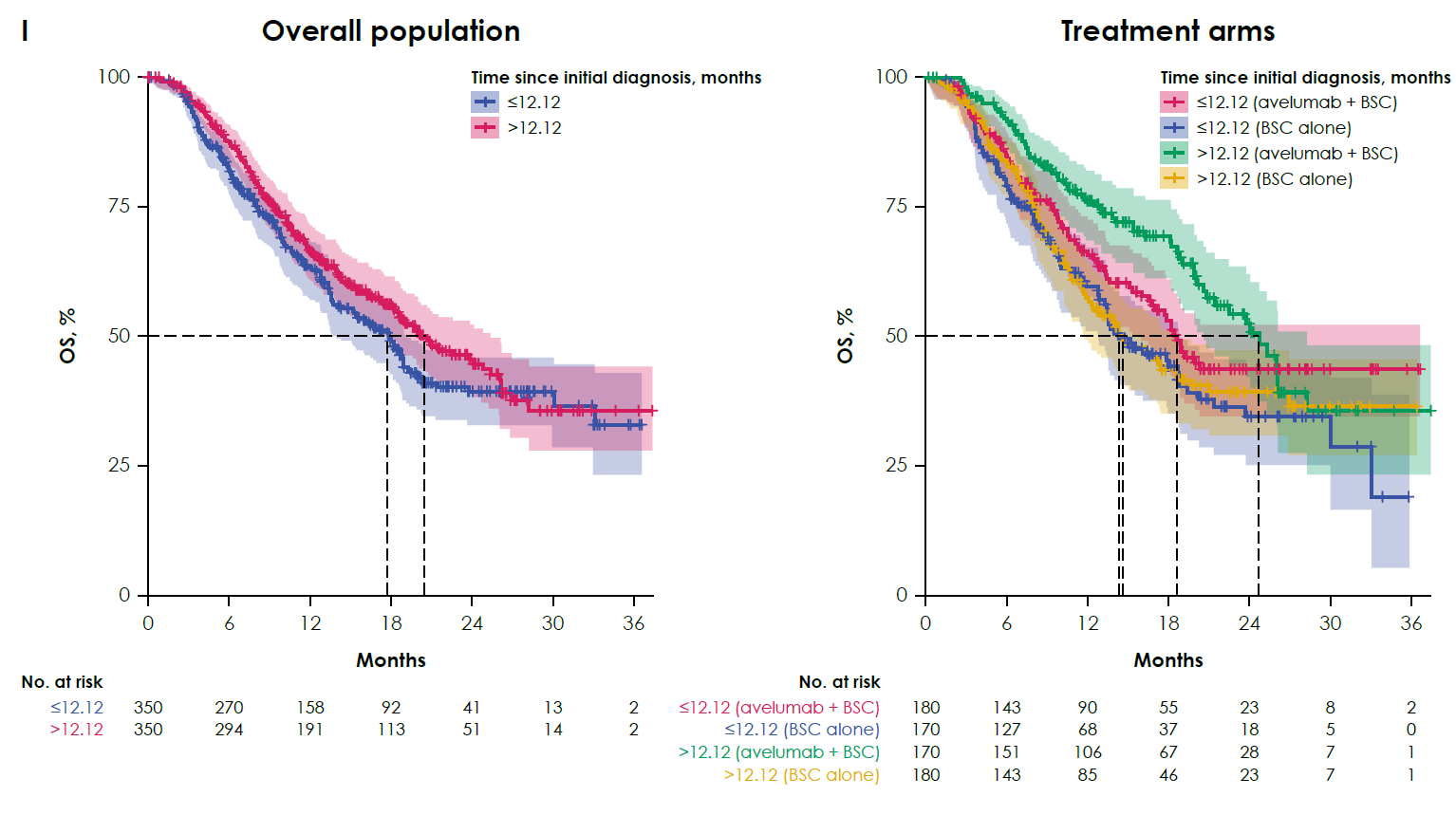
**

1. **Visceral metastases**

**
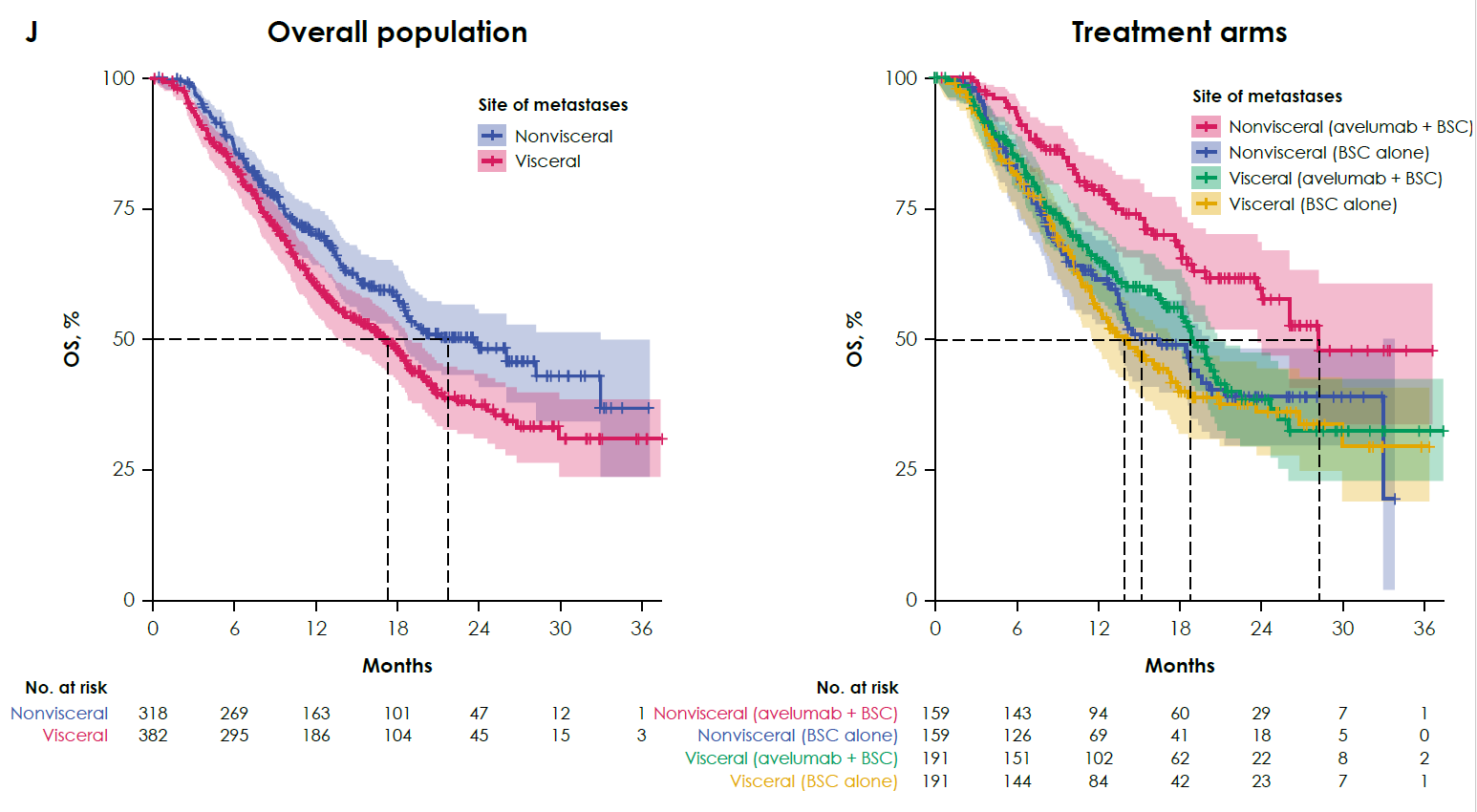
**

1. **Age**

**
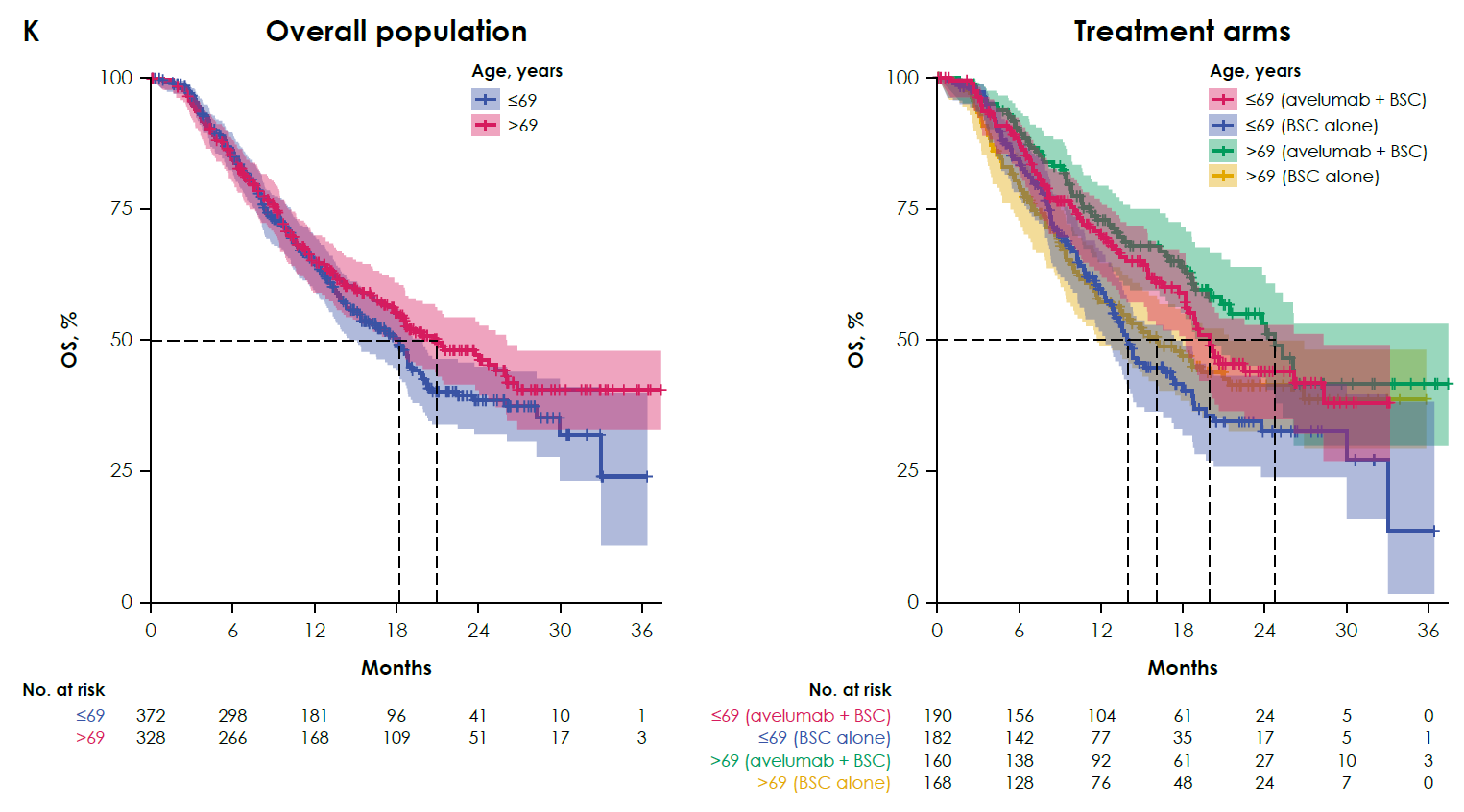
**

1. **PD-L1 expression**

**
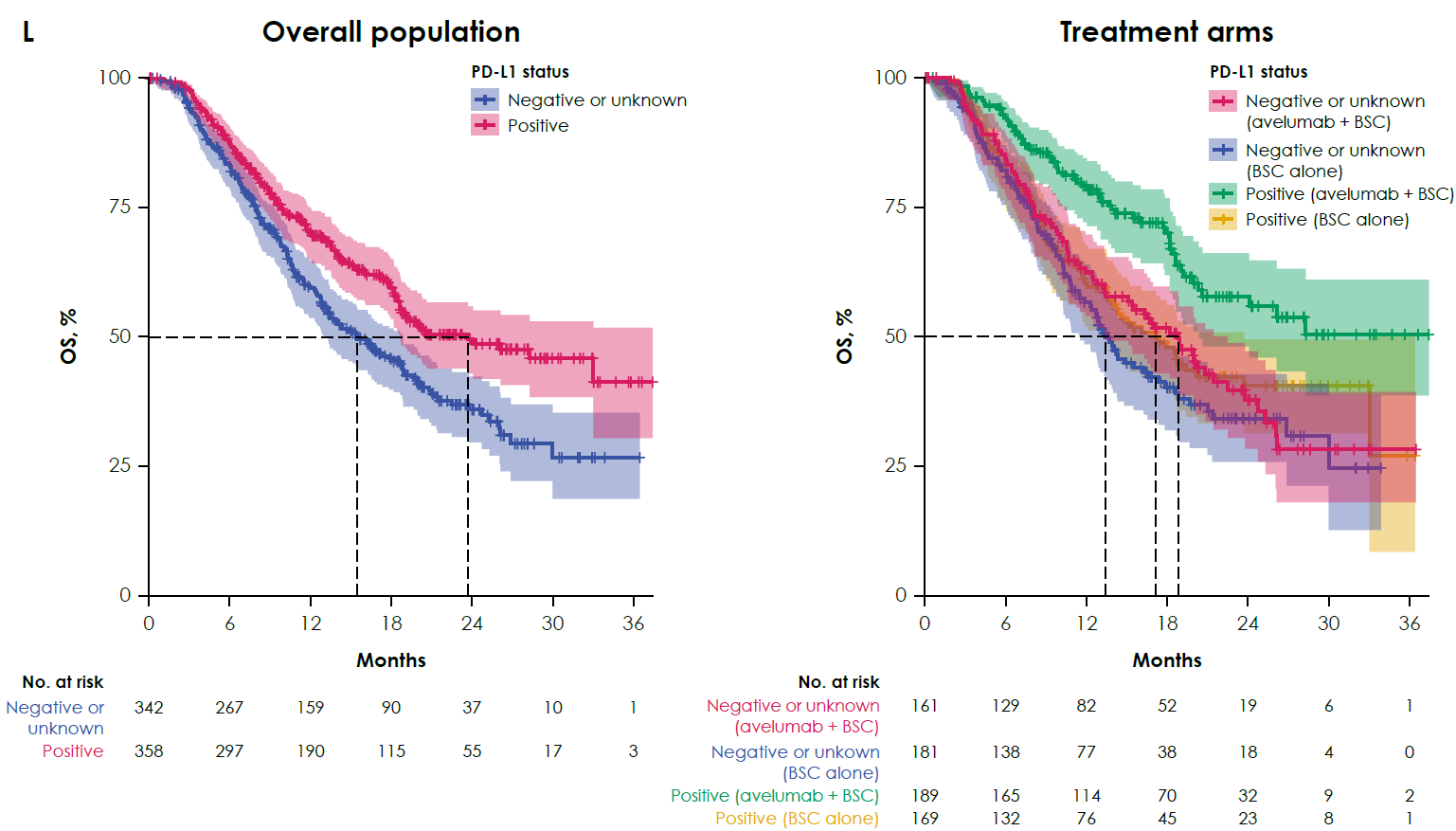
**
